# Acute Lung Injury Biomarkers in the Prediction of COVID-19 Severity: Total Thiol, Ferritin and Lactate Dehydrogenase

**DOI:** 10.3390/antiox10081221

**Published:** 2021-07-29

**Authors:** Alvaro Martinez Mesa, Eva Cabrera César, Elisa Martín-Montañez, Esther Sanchez Alvarez, Pilar Martinez Lopez, Yanina Romero-Zerbo, Maria Garcia-Fernandez, Jose Luis Velasco Garrido

**Affiliations:** 1 Servicio de Neumología, Hospital Universitario Virgen de la Victoria, 29010 Málaga, Spain; alvaro.martinez.sspa@juntadeandalucia.es (A.M.M.); esther.sanchez.sspa@juntadeandalucia.es (E.S.A.); josel.velasco.sspa@juntadeandalucia.es (J.L.V.G.); 2 Departamento de Fisiología Humana, Facultad de Medicina, Instituto de Investigación Biomédica de Málaga, Universidad de Málaga, 29010 Málaga, Spain; yaninaromero@uma.es (Y.R.-Z.); igf@uma.es (M.G.-F.); 3 Departamento de Farmacología y Pediatría, Facultad de Medicina, Instituto de Investigación Biomédica de Málaga, Universidad de Málaga, 29010 Málaga, Spain; emartinm@uma.es; 4 Unidad de Cuidados Intensivos, Hospital Universitario Virgen de la Victoria, 29010 Málaga, Spain; pilar.martinez.sspa@juntadeandalucia.es

**Keywords:** COVID-19, acute respiratory distress syndrome, biomarkers, total thiol, ferritin, LDH, prognosis

## Abstract

SARS-CoV-2 (COVID-19) patients who develop acute respiratory distress syndrome (ARDS) can suffer acute lung injury, or even death. Early identification of severe disease is essential in order to control COVID-19 and improve prognosis. Oxidative stress (OS) appears to play an important role in COVID-19 pathogenesis; we therefore conceived a study of the potential discriminative ability of serum biomarkers in patients with ARDS and those with mild to moderate disease (non-ARDS). 60 subjects were enrolled in a single-centre, prospective cohort study of consecutively admitted patients: 29 ARDS/31 non-ARDS. Blood samples were drawn and marker levels analysed by spectrophotometry and immunoassay techniques. C-reactive protein (CRP), lactate dehydrogenase (LDH), and ferritin were significantly higher in ARDS versus non-ARDS cases at hospital admission. Leukocytes, LDH, ferritin, interleukin 6 (IL-6) and tumour necrosis factor alpha (TNF-α) were also significantly elevated in ARDS compared to non-ARDS patients during the hospital stay. Total thiol (TT) was found to be significantly lower in ARDS. Conversely, D-dimer, matrix metalloproteinase-9 (MMP-9) and advanced glycosylated end products (AGE) were elevated. Leukocytes, LDH, CRP, ferritin and IL-6 were found to be significantly higher in non-survivors. However, lymphocyte, tumour necrosis factor beta (TGF-β), and TT were lower. In summary, our results support the potential value of TT, ferritin and LDH as prognostic biomarkers for ARDS development in COVID-19 patients, distinguishing non-ARDS from ARDS (AUCs = 0.92; 0.91; 0.89) in a fast and cost-effective manner. These oxidative/inflammatory parameters appear to play an important role in COVID-19 monitoring and can be used in the clinical management of patients.

## 1. Introduction

Coronaviruses (CoVs) have been the cause of respiratory tract infections for over 50 years. Previous epidemic CoV outbreaks include severe acute respiratory syndrome (SARS)-CoV and Middle East respiratory syndrome (MERS)-CoV [1]. The new coronavirus disease produced by SARS-CoV-2 (COVID-19), causing a wide range of effects from non-symptomatic, mild to moderate disease, to severe infection resulting in fatal disease, has been a threat to public health since the end of 2019. While many patients experience only a mild form, severe illness develops in 14% of cases, and critical illness in 5%, with acute respiratory distress syndrome (ARDS), multiple organ failure, and even death [2,3].

The major risk factors associated with severe COVID-19 are increasing age and comorbidities, cardiovascular disease, diabetes and especially respiratory disease [4,5]. While COVID-19 pathogenesis has not yet been characterised, there is evidence in the literature pointing to possible mechanisms.

10% to 20% of patients with a severe form of the disease develop lung failure due to severe ARDS, which is associated with high morbidity and mortality [3]. The occurrence of an exaggerated inflammatory immune response in severe and fatal COVID-19 patients, characterised by excessive systemic elevation of several pro-inflammatory cytokines [6,7], is already known. The cytokine storm may be a driver behind lung injury and ARDS, oedema and lung fibrosis at later stages.

In fact, patients who develop ARDS can suffer acute lung injury and extracellular matrix (ECM) remodelling [8]. The lung lesion and its reparation in COVID-19 patients exhibit many similarities with onset and progression of interstitial lung diseases (ILD). Thus, expression of pro-fibrotic markers, which are elevated in ILD, could be altered in COVID-19 patients who develop ARDS, and result in pulmonary fibrosis.

With regard to haematology-associated complications of COVID-19, coagulation markers such as D-dimer are also elevated in patients with both severe and fatal disease [9,10], and thrombosis is primarily associated with inflammation [11,12].

Additionally, oxidative stress (OS) appears to have an important role in COVID-19 pathogenesis [13,14]. A common factor in all the aforementioned conditions associated with COVID-19 seems to be the impaired redox homeostasis responsible for the accumulation of reactive oxygen species (ROS). Moreover, OS develops as a result of ROS and the depletion of antioxidant mechanisms, playing an important role in viral replication and the pathogenesis of subsequent virus-associated diseases [15]. An example of this is the state of hyper-coagulability, since it causes endothelial dysfunction and damage to endothelial cell lining [16]. Early identification of severe COVID-19 is critical to control the disease and improve the prognosis. The identification of laboratory predictors for progression towards severe and fatal forms of this illness is needed. In this study we set out to evaluate the potential discriminative ability of haematologic, inflammatory and coagulation parameters, together with pro-fibrotic and oxidative stress markers, in patients who develop ARDS, and patients with mild to moderate disease.

## 2. Materials and Methods

### 2.1. Study Design and Patients

This was a single-centre, prospective cohort study of patients diagnosed with COVID-19, consecutively admitted to the ‘Virgen de la Victoria University Hospital’, a tertiary hospital in Malaga, Spain, over two months. The hospital serves a population of 500,000 in the greater Malaga area. Eligible for inclusion were 60 patients ≥18 years old with a positive SARS-CoV-2 real-time reverse-transcriptase-polymerase chain reaction (rRT-PCR), cobas^®^ test, from nasopharyngeal swab, sputum or bronchoalveolar lavage and pneumonia or ARDS symptoms. Non-eligible patients were those who were positive rRT-PCR but asymptomatic or symptomatic without a radiological image compatible with pneumonia or ARDS. The patients were divided into two groups: ARDS patients (severe patients) and those with mild to moderate pneumonia (non-ARDS group). The diagnosis of ARDS was made in the Emergency Department or during the first 24–48 h of stay in the hospital ward. ARDS is defined as that situation or situations that alter gas exchange, (A) with a relationship between the arterial partial pressure of oxygen and the fraction of inspired oxygen (PaO_2_/FiO_2_) less than 200, (B) bilateral infiltrates in both lung fields in the chest X-ray, and (C) pulmonary wedge pressure (PCP) less than 18 mmHg [17].

Study methods were conducted according to the Declaration of Helsinki and approved by the Ethics in Human Research Committee of Malaga University Hospital on 7 May 2020, code 01-2020. Informed consent was obtained from all participants. At diagnosis, and after providing informed consent, a blood sample was drawn from a vein (veni-puncture). A week after hospital admission a second sample was collected (monitoring analysis). Information about demographic data (age, sex), smoking habits and clinical variables was gathered from the clinical history.

### 2.2. Sample Preparation and Marker Measurements

Whole blood was collected in tubes containing EDTA at a final concentration of 50 mM and immediately processed to measure routine laboratory markers. For plasma preparation to measure specific markers, blood was centrifuged at 1200 g for 10 min at 4 °C and the supernatant was stored in siliconized tubes at −80 °C until use. The main laboratory findings for each patient (blood cells, lactate dehydrogenase -LDH-, C-reactive protein -CRP-, D-dimer and ferritin) are taken routinely. The following markers IL-6, IL-7, MMP-1, MMP-9, TGF-β, TNF-α and TNF-β were measured by ProcartaPlex multiplex immunoassay (Thermo Fisher Scientific, Waltham, MA, USA).

Levels of lipid hydroperoxides (LOOH) and sulfhydryl (-SH) groups, as total thiol (TT), were measured by spectrophotometry using methods adapted to an ICubio AutoAnalyzer. LOOH level was calculated relative to a hydrogen peroxide standard curve and was expressed as nmol/mg of protein using the FOX2 method (Roche, Basel, Switzerland). TT was determined using Ellman’s reagent 5,5′-dithiobis (2-nitrobenzoate)-DTNB [18] and -SH concentration was calculated using a standard curve of glutathione [19].

Levels of advanced glycosylated end products (AGE) and lysophosphatidic acid (LPA) were assessed by enzyme-linked immunosorbent assay (ELISA) using commercially available ELISA kits (OxiSelect™ AGE ELISA Kit, Cell Biolabs, Inc., San Diego, CA, USA; LPA EA-kit K-2800S, Echelon Biosciences Inc., Salt Lake City, UT, USA). Finally, the human receptor for AGE (RAGE) was measured by a commercial sandwich-type ELISA Kit (RAGE/AGER ELISA Kit, cat. number CSB-E09354h, Cusabio, Houston, TX, USA). All assays were carried out according to the manufacturers’ instructions.

### 2.3. Statistical Analysis

The variables were expressed as the mean (±SD) or n (%). To compare quantitative measures or time series, a *t*-test or Mann-Whitney U-test and Wilcoxon rank test was used, as appropriate. A chi-squared test was used for qualitative comparison. For bivariate correlation, the Rho Sperman correlation coefficient was used. In order to compute the sensitivity and specificity of the variables, receiver operating characteristic (ROC) curves were calculated. In order to evaluate the performance of the serum markers as prognostic markers, the area under the curve (AUC) of the receiver operating characteristics (ROC) curve was calculated. The cut-off value of serum markers was determined using the Youden index. A significance of 5% (*p* < 0.05) was required to consider a difference to be statistically significant. Statistical analysis was performed using SPSS software version 22.0 (IBM, Chicago, IL, USA).

## 3. Results

Sixty consecutive SARS-CoV-2 positive adult patients admitted to the hospital were included (Table 1). Of these, 29 patients (48.3%) were diagnosed with ARDS. Although 21 (72.4%) were admitted to the intensive care unit (ICU), 8 (27.6%) were not hospitalised in the ICU due to their comorbidities (ARDS group).

At the time of obtaining the follow up sample of the patients admitted to the ICU, 8 (38.1%) received invasive mechanical ventilation (the minimum positive end-expiratory pressure used was 5 cmH_2_O, and 10 cmH_2_O for severe cases); the rest received high-flow oxygen. The data in Table 1 show the respiratory support during the entire admission. In most cases, high flow nasal cannula was used as first respiratory support or to facilitate weaning from invasive mechanical ventilation.

31 patients (51.6%) were diagnosed with mild to moderate pneumonia (non-ARDS group). Demographic data and clinical characteristics are shown in Table 1. Similar ages and percentages of comorbidities were found in these two groups, whereas the percentages of male, and active or former smokers in ARDS patients, were statistically significantly higher than those in the non-ARDS group (*p* = 0.019); moreover, women presented non-ARDS more frequently (*p* = 0.014). No differences were found between men and women for any parameter. Concerning final outcomes, length of hospital stays tripled in ARDS patients (*p* = 0.000), and exitus was significantly elevated in the ARDS group compared to non-ARDS patients (*p* = 0.049). With respect to the treatments, at the time the samples were taken, 51 patients (85%) received antiviral treatment with Lopinavir/Ritonavir, 56 (93.3%) Hydroxychloroquine, 12 (20%) Azithromycin, 40 (66.7%) systemic corticosteroids, 23 (38.3%) Interferon and 23 (38.3%) Tocilizumab. It should be borne in mind that these therapies were assigned based on the initial knowledge of COVID-19, with no differences in the treatments administered to ARDS vs. non-ARDS patients.

Routine laboratory marker values on hospital admission are shown in Table 2. In a comparison of the ARDS group with mild to moderate pneumonia patients, there were no significant differences in blood cells and D-dimer, while LDH, CRP and ferritin were significantly higher among ARDS patients than in the non-ARDS group (*p* < 0.05 for all). A week after admission, in a control analysis (Table 3), leukocytes and D-dimer values were statistically significantly higher than in the non-ARDS group (*p* = 0.001 for both), and LDH and ferritin levels remained higher (*p* = 0.04 and *p* = 0.001, respectively).

The measurement of the specific markers AGE, RAGE, IL-6, IL-7, LOOH, LPA, MMP-1, MMP-9, TGF-β, TNF-α, TNF-β and TT detected statistically significant differences in AGE/RAGE, IL-6, MMP-9, TNF-α and TT between groups (Table 3). Furthermore, AGE, IL-6, MMP-9 and TNF-α values were significantly elevated in ARDS compared to non-ARDS patients. Conversely, TT and RAGE values were found to be significantly lower in ARDS patients (*p* = 0 and *p* = 0.001, respectively).

On the analysis of serum marker levels among patients discharged alive and those who died; leukocytes, LDH, CRP, ferritin and IL-6 values were found to be significantly higher in non-survivors (Table 4). However, lymphocyte, TGF-β and TT levels were significantly lower than in patients discharged alive (*p* = 0.031, *p* = 0.009 and *p* = 0.002, respectively).

Applying a multiple linear regression to each of the biomarker levels, adjusting for gender and smoking status, we found that only for IL-6 and ferritin does the smoking variable add statistically significant differences to the prediction, *p* < 0.05. There were no statistically significant differences to the prediction by gender (Supplementary Table S1).

We also tested the correlation coefficient to study the relationship between serum levels of TT and those variables that were statistically significant in Table 3 (Supplementary Table S2). Serum levels of LDH (admission), Leukocyte count (1 week), D-dimer (1 week), IL-6, TNF-α and RAGE showed a negative correlation with serum TT.

**Figure 1:**
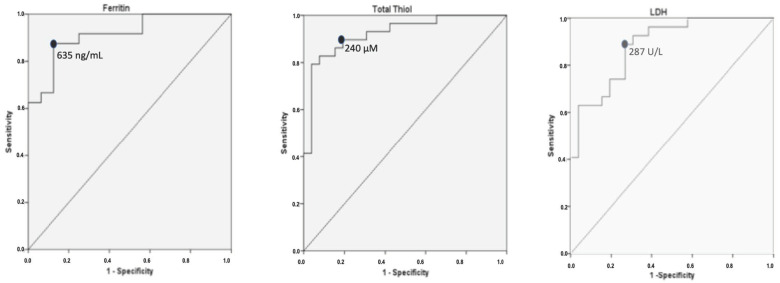
Areas under the curves and cut-off values of ferritin, total thiol and LDH in the distinction between patients who develop acute respiratory distress syndrome and patients with mild to moderate disease.

The ROC analysis for LDH, CRP, ferritin, leukocytes, D-dimer, AGE, IL-6, MMP-9, TNF-α and TT are given in Table 5. According to the results, TT, ferritin and LDH are the best parameters to distinguish mild to moderate COVID-19 patients (non-ARDS group) from severe patients (ARDS group) followed by IL-6 and D-dimer. The largest AUCs were found for TT and ferritin a week after admission (AUC = 0.92; 95% CI 0.85–0.99 *p* = 0; AUC = 0.91; 95% CI 0.83–1 *p* = 0, respectively) and for LDH on hospital admission (AUC = 0.90; 95% CI 0.83–0.98 *p* = 0). The best cut-off values chosen for ferritin, TT and LDH to predict a poor clinical course were 635 ng/mL (sensitivity: 87.5%, specificity: 81.2%), 240 uM (sensitivity: 86%, specificity: 85%) and 287 U/L (sensitivity: 89%, specificity: 73%), respectively (Figure 1). In the distinction of patient groups using IL-6 and D-dimer the AUCs were found to be 0.87 (95% CI 0.79–0.96; *p* = 0), and 0.86 (95% CI 0.75–0.96; *p* = 0), respectively, while the AUCs for leukocytes and MMP-9 were found to be 0.85 and 0.82 (leukocytes: 95% CI 0.75–0.95; *p* = 0; MMP-9: 95% CI 0.71–0.93; *p* = 0) (Supplementary Figure S1a–i).

## 4. Discussion

Previous studies have shown the relation between haematologic, inflammatory and coagulation parameters with the severity of the disease [20,21,22,23,24], although only a limited number of studies examine the effect of pro-fibrotic and OS markers on the severe clinical course of COVID-19 patients. In this study we analysed, for the first time, the potential value of haematologic, inflammatory and coagulation parameters, together with pro-fibrotic and OS markers as prognostic biomarkers for ARDS development in COVID-19 patients. We selected these biomarkers from the existing literature [25], and also new ones in relation to the role that OS appears to play in the pathogenesis of COVID-19. ROS can break the lipid membrane and increase fluidity and membrane permeability of endothelial cells, RBCs, platelets and leukocytes, and all of these phenomena are altered by COVID-19 [26]. ROS also produces protein damage, peptide chain fragmentation, cross-linked reaction product aggregation, electric charge alteration, enzymatic inactivation, and proteolysis susceptibility. All these factors cause, on the one hand, endothelial dysfunction and damage to endothelial cell lining, activating platelets and leukocytes, consequently affecting the clotting system, and can also directly cause structural defects in molecules that could increase procoagulant activity, contributing to systemic coagulation activation.

We compared the serum concentrations of these compounds in severe COVID-19 patients (ARDS group) and those with mild to moderate pneumonia (non-ARDS group). Our data show elevated serum levels of CRP, LDH and ferritin in all COVID-19 patients, which are significantly higher in the ARDS group than in non-ARDS patients on hospital admission. Moreover, leukocyte, LDH, ferritin, IL-6 and TNF-α values were also significantly elevated in ARDS compared to non-ARDS patients a week after admission, indicating the presence of high systemic inflammation. These elevated inflammatory marker values, together with a decrease in TT as an antioxidant defence in the human body, lymphocytes and the pro-fibrotic factor TGF-β, may lead to increased mortality in hospitalised COVID-19 patients; TT, ferritin and LDH are shown to be the best markers for predicting disease severity. D-dimer, MMP-9 and AGE values were found to be significantly elevated in severe patients during their hospital stay.

The haematological abnormalities found in patients who develop severe (ARDS group) and fatal (non-survivors) COVID-19, are similar to the findings of the meta-analysis carried out by Henry et al. [9], where patients with severe and fatal disease had significantly increased leukocyte counts and decreased lymphocyte counts when compared to patients with non-severe disease and survivors.

With respect to the inflammatory biomarkers analysed (CRP, LDH, ferritin, IL-6, IL-7, TNF-α and TNF-β), significantly greater increases were found for routine markers and some cytokines in patients with worse evolution. Ferritin was one of the first serum markers to be related to poor prognosis. Elevated ferritin levels were observed in non-surviving Wuhan patients [4]. The significantly elevated levels of ferritin found in ARDS group on hospital admission, maintained during the hospital stay, together with the significant increase of ferritin levels in non-survivors vs. survivors, and the fact that ferritin was one of the best parameters for distinguishing non-ARDS patients from severe patients, lead to the suggestion of ferritin as a high-sensitivity biomarker for monitoring COVID-19 patients at hospital admission and over the course of hospitalisation. Ferritin elevation, along with the significantly raised CRP levels on hospital admission, point to the development of ARDS. Another study proposed CRP as an early predictive marker of severe COVID-19 [20]. CRP in severe COVID-19 patients increased significantly at the initial stage, before computed tomography findings.

With respect to inflammatory cytokines, significantly higher IL-6 and TNF-α values were found in severe patients (ARDS group) compared to non-ARDS patients, a week after admission; these are considered significant predictors of disease severity and death [27]. The exaggerated elevation of interleukins, which can lead to a cytokine storm, may be a driver behind acute lung injury and ARDS, and lead to new tissue damage progressing to multiple organ failure [28]. Moreover, the ROC analysis showed that the ability of the AUC of IL-6 to predict disease severity was one of the highest, demonstrating its predictive power for the severity of COVID-19 patients over the course of hospitalisation. In fact, determining the IL-6 level, together with TNF-α, could be considered as a clinical tool for stratifying high-risk patients, and identifying those who should be treated with the IL-6R antagonist tocilizumab [21,22,23,24,27,28,29]. IL-7, a cytokine with a similar signal transduction pathway to IL-6, may be considered a prognostic marker [30,31]. However, a significant relationship between IL-7 values and the clinical course of the disease has not so far been found.

The cytokine release syndrome and the immune thrombosis induced by SARS-CoV-2 could explain the severity of COVID-19 [32]. The levels of D-dimer as a coagulation parameter were statistically significantly higher in patients who developed ARDS than in mild to moderate pneumonia patients. Data in the literature frequently show enhanced D-dimer values in COVID-19 patients (36–43% of positive cases) [9] and their use in evaluating the prognosis of COVID-19 patients has been proposed [24]. The AUC of D-dimer found in our study provides support for this use.

The present study shows that CRP, LDH, ferritin and IL-6 are related not only with COVID-19 severity but also with mortality. These parameters were statistically increased in fatal patients. A retrospective multi-centre study also found an increase of these parameters in non-survivors compared to survivors [33]. Conversely, the pro-fibrotic factor and anti-inflammatory TGF-β was statistically decreased in non-surviving patients, which is a significant finding due to the consideration of TGF-β blockade as a potential treatment in COVID-19 patients [34]. The SARS-CoV-2 viral infection and the consequent strong immune and inflammatory response could induce the massive increase in active TGF-β in the lungs, and the decrease in its circulating levels. Our result would then support this hypothesis. The decrease in circulating TGF-β should alert clinicians to a possible massive lung activation, suggesting poor prognosis and a new therapeutic approach. Matrix metalloproteinase (MMP) MMP-9, a pro-fibrotic marker which is elevated in patients who develop ARDS [35], was significantly elevated in the ARDS group in our study. In fact, this neutrophil activation-associated biomarker may be increased in COVID-19 patients with more severe disease, showing positive correlation with the risk of death [36]. MMPs are dysregulated during the inflammatory phase of ARDS, degrading ECM proteins and causing epithelial and endothelial injury [37]. They are released and activated in inflammatory and OS conditions [38].

Oxidative stress plays a key role in the development of lung fibrosis and cardiovascular complications, the need for mechanical ventilation, and mortality in COVID-19 patients [39].The use of antioxidant molecules in its treatment, and their clinical benefits, has been studied [23,40,41,42]. Some studies have proposed N-acetylcysteine (NAC) as a possible treatment due to its antioxidant, anti-inflammatory and immune-modulating characteristics, which could be beneficial in the treatment and prevention of SARS-CoV-2 [43].

Redox homeostasis seems to be strongly altered in severe COVID-19 patients, showing a decrease in antioxidant molecules that neutralise oxidative molecules [14,44] and an increase in oxidative damage in cell structures such as lipids, membranes, proteins, and nucleic acids [45]. Also, a recent study described a model in which the increase in OS at the cellular level would favour the entry of different types of viruses, with SARS-CoV and SARS-CoV-2 being especially important [46].

In our study, levels of the antioxidant TT were significantly lower in severe patients (ARDS group) compared to the non-ARDS group, giving the best AUC for predicting disease severity. TT was also statistically decreased in fatal patients. These results demonstrate the excellent predictive power of TT in determining the clinical severity of COVID-19 over the duration of hospitalisation and supports the use of thiol-based antioxidants as clinical tools for stratifying high-risk patients, as fast and cost-effective tests [14]. We observe in our data that lower levels of thiols are related to an increase in hypercoagulability, D-dimer, and IL-6, all of which contribute directly to greater inflammation, an alteration in coagulation, and stress, coinciding with a worse clinical evolution.

Thiol measurement by automated spectrophotometry was also shown to be cheaper and much faster (<10 min) compared to the IL-6 test.

Regarding the biomarker of lipid peroxidation, LOOH, no significant differences were detected between COVID-19 patients. The study of Pincemail et al. showed an increase in lipid peroxidation in COVID-19 patients hospitalised in the ICU compared to reference value [45]. They state that, although the elevated levels of lipid peroxides found were not statistically different from the reference interval, a majority of COVID-19 patients (63.6%) exhibited higher levels of hydroperoxides than the upper reference value. By contrast, ox-LDL, as another form of lipid peroxidation, did not confirm the presence of increased oxidative damage to lipids. Therefore, new studies using biomarkers of lipid peroxidation need to be carried out.

Advanced glycosylated end products, which are formed by a combination of glycation, oxidation and/or carbonylation, are accumulated in aging and inflammatory diseases, but also in situations of OS overload. They lead to excessive accumulation of ECM and expression of pro-fibrotic markers e.g., TGF-β [47]. The AGE/RAGE axis is associated with lung processes such as IPF or severe ARDS [48,49,50,51,52] at several mechanisms [53]. In this study, whereas AGE levels were found to be significantly elevated in the ARDS group, RAGE was found to be significantly lower. In this sense, Reynolds et al. [54] found a delay in the development of lung injury and mortality in mice with RAGE-null expression. Our results provide support for the suggested important role of the RAGE pathway in the aggravation of COVID-19 [55]. This theory is based on high RAGE levels found in young asymptomatic COVID-19 patients versus the low RAGE levels in elderly patients with lung involvement. However, the intrinsic limits of that biomarker must be taken into account [53]. Therefore, the AGE/RAGE axis could be considered to play an important clinical role in ARDS pathogenesis in COVID-19 and the use of RAGE pathway modulators must be considered as a possible treatment for the reduction of morbidity and mortality.

This study is one of the few published in the literature that relates OS biomarkers with the evolution of COVID-19, and so there will be options for new patient management and new lines of treatment.

We are aware that our study has some limitations, such as the low number of patients, and limited external validity, due to being only a single-centre study. Multicentre studies would be needed to confirm these results. It would also be interesting to compare these groups with other populations that develop ARDS.

## 5. Conclusions

Our study analyses, for the first time, the potential value of haematologic, inflammatory and coagulation parameters, together with pro-fibrotic and OS markers as prognostic biomarkers for ARDS development in COVID-19 patients. Early identification of severe COVID-19 is critical to control the disease and improve the prognosis, and the identification of laboratory predictors for progression towards severe and fatal forms of this illness is essential. Our results provide support for the potential use of TT, ferritin, LDH, IL-6 and D-dimer as prognostic biomarkers for the development of ARDS, showing TT, ferritin and LDH to be the most valuable. The relevance of MMP-9, the AGE/RAGE axis, leukocytes, CRP and TNF-α is unclear, but certainly warrants further research. Moreover, TT, CRP, LDH, leukocytes, ferritin and IL-6 would be related not only with COVID-19 severity but also with mortality, in the same way as lymphocytes and TGF-β. The increase in these haematologic, inflammatory biomarkers, or a decrease in TT, lymphocytes and TGF-β, may lead to increased mortality. Therefore, the value of these biomarkers in predicting mortality needs to be considered and further explored. Knowledge of all these findings may lead to the development of new therapeutic approaches. In conclusion, changes in these serum biomarkers, mainly the highly sensitive markers TT, ferritin and LDH, may play an important role in COVID-19 monitoring, and they can also be used in the clinical management of patients. Thus, we suggest the use of the routine laboratory markers ferritin and LDH and the fast and cost-effective thiol-based antioxidant biomarkers to distinguish patients who develop ARDS from patients with mild to moderate disease.

## Figures and Tables

**Table 1:** Baseline demographic and clinical characteristics and final outcomes of the patients who developed acute respiratory distress syndrome, or pneumonia without this criterion.

Characteristics	ARDS	Non-ARDS	*p* Value
**Patients n**	29	31	
Age, years	67 ± 13	64 ± 9	NS
Male	23 (79.3)	15 (48.4)	0.01
Female	6(20.7)	16 (51.6)	0.01
**Smoking status**			
Active or former smokers	15 (51.7)	7 (11.6)	0.019
**Comorbidities**			
Arterial hypertension	16 (55.2)	15 (48.4)	NS
Diabetes mellitus	11 (37.9)	10 (32.3)	NS
Dyslipidaemia	12 (41.4)	12 (38.7)	NS
Heart disease	4 (13.8)	2 (6.4)	NS
Pulmonary disease	3 (10.3)	4 (12.9)	NS
**Previous treatment**			
ACE blocker drugs	14 (48.3)	13 (31.9)	NS
Inhaled corticoids	1 (3.4)	4 (12.9)	NS
**SOFA score**	3.7 ± 2.5	1.1 ± 1.2	0.000
**Respiratory support**			
Invasive mechanical ventilation	23 (79.3)	0 (0.0)	0.000
High Flow Nasal Cannula	8 (27.6)	2(6.9)	0.028
Length of IMV	22.9 ± 13.9	-	-
**Final outcomes**			
Hospitalisation in ICU	21 (72.4)	1 (3.2)	0.000
Length of hospital stay (days)	40.9 ± 25.3	12.65 ± 7.3	0.000
Exitus	5 (17.2)	2 (6.4)	0.049

Continuous variables are expressed as the mean (±SD). Categorical data are expressed as n (%). ARDS: Acute respiratory distress syndrome. ACE: Angiotensin-converting enzyme. ICU: Intensive care unit. NS: non-significant. IMV: Invasive Mechanical Ventilation. SOFA: Sepsis related Organ Failure Assessment.

**Table 2:** Values of variables on hospital admission.

Variables	ARDS	Non-ARDS	*p* Value
**Patients n**	29	31	
**Blood cells count**			
Leukocyte count (×10^9^/L)	6.081 ± 2.372	7.172 ± 2.617	NS
Lymphocyte count (×10^9^/L)	0.988.51 ± 0.465	1.221 ± 0.555	NS
**Routine markers**			
LDH (U/L)	463 ± 179	269 ± 61	0.000
CRP (mg/L)	143.6 ± 91.1	77.6 ± 61.2	0.002
D-dimer (ng/mL)	1136 ± 856	1260 ± 1041	NS
Ferritin (ng/mL)	1226 ± 608	540 ± 451	0.002

Values are expressed as the mean (±SD). ARDS: Acute respiratory distress syndrome. LDH: Lactate dehydrogenase. CRP: C-reactive protein. NS: non-significant.

**Table 3:** Values of variables a week after hospital admission.

Variables	ARDS	Non-ARDS	*p* Value
**Patients n**	29	31	
**Blood cells count**			
Leukocyte count (×10^9^/L)	10.674 ± 3.398	6.550 ± 2.248	0.000
Lymphocyte count (×10^9^/L)	1.371 ± 0.908	1.566 ± 0.741	NS
**Routine markers**			
LDH (U/L)	330 ± 104	259 ± 60	0.002
CRP (mg/L)	77.3 ± 62.1	49.2 ± 63.7	NS
D-dimer (ng/mL)	3648 ± 999	1216 ± 1233	0.000
Ferritin (ng/mL)	1226 ± 608	540 ± 451	0.000
**Specific markers evaluated**			
AGE (ng/mL)	20.88 ± 5.3	15.74 ± 4.7	0.000
RAGE (ng/L)	147.8 ± 120.6	504.50 ± 262.8	0.001
IL-6 (pg/mL)	260.5 ± 117.5	22.2 ± 22.3	0.000
IL-7 (pg/mL)	166.2 ± 174.4	151.6 ± 213.7	NS
LOOH (μM)	75.0 ± 4.2	76.9 ± 4.4	NS
LPA (nM)	142.9 ± 75.7	149.2 ± 74.3	NS
MMP-1 (μg/L)	2568 ± 1242	2519 ± 1817	NS
MMP-9 (μg/L)	236.4 ± 52.2	130.4 ± 91.8	0.000
TGF-β (ng/mL)	504.3 ± 187.7	481.5 ± 185.6	NS
TNF-α (ng/L)	20.89 ± 30.54	8.58 ± 1.20	0.029
TNF-β (ng/L)	16.24 ± 24.74	13.22 ± 17.09	NS
TT (μM)	203.4 ± 40.2	301.8 ± 73.1	0.000

Values are expressed as the mean (±SD). ARDS: Acute respiratory distress syndrome. LDH: Lactate dehydrogenase. CRP: C-reactive protein. AGE: Advanced glycosylated end products. RAGE: the human receptor for AGE. IL-6: Interleukin 6. IL-7: Interleukin 7. LOOH: Lipid hydroperoxides. LPA: lysophosphatidic acid. MMP-1: Matrix metalloproteinase-1. MMP-9: Matrix metalloproteinase-9. TGF-β: Transforming growth factor beta. TNF-α: Tumour necrosis factor alpha. TNF-β: Tumour necrosis factor beta. TT: Total thiol. NS: non-significant.

**Table 4:** Values of variables a week after hospital admission of the patients who survived and those who did not survive.

Variables	Exitus	Alive	*p* Value
**Patients n**	7	53	
**Blood cells count**			
Leukocyte count (×10^9^/L)	11.377 ± 1.623	8.053 ± 3.315	0.015
Lymphocyte count (×10^9^/L)	1.053 ± 0.506	1.503 ± 0.833	0.031
**Routine markers**			
LDH (U/L)	402 ± 89	274± 78	0.001
CRP (mg/L)	116.5 ± 19.1	55.7 ± 63.9	0.048
D-dimer (ng/mL)	3022 ± 999	1989 ± 1760	NS
Ferritin (ng/mL)	1666 ± 1217	779 ± 476	0.038
**Specific markers evaluated**			
AGE (ng/mL)	20.2 ± 5.4	18.0 ± 5.8	NS
RAGE (ng/L)	202.0 ± 219.6	357.8 ± 277.7	NS
IL-6 (pg/mL)	459.0 ± 604.3	104.6 ± 252.3	0.008
IL-7 (pg/mL)	154.0 ± 195.6	160.3 ± 199.1	NS
LOOH (μM)	75.5 ± 4.0	76.2 ± 4.4	NS
LPA (nM)	126.8 ± 76.6	151.3 ± 73.4	NS
MMP-1 (μg/L)	2164 ± 619	2604 ± 1659	NS
MMP-9 (μg/L)	159.2 ± 98.2	180.1 ± 92.8	NS
TGF-β (ng/mL)	371.2 ± 105.7	511.9 ± 191.0	0.009
TNF-α (ng/L)	21.66 ± 31.07	19.92 ± 21.33	NS
TNF-β (ng/L)	9.83 ± 0.75	9.97 ± 22.50	NS
TT (μM)	156.2 ± 48.5	266.6 ± 73.9	0.002

Values are expressed as the mean (±SD). Exitus: non-surviving patients. Alive: patients discharged from the hospital. ARDS: Acute respiratory distress syndrome. LDH: Lactate dehydrogenase. CRP: C-reactive protein. AGE: Advanced glycosylated end products. RAGE: the human receptor for AGE. IL-6: Interleukin 6. IL-7: Interleukin 7. LOOH: Lipid hydroperoxides. LPA: lysophosphatidic acid. MMP-1: Matrix metalloproteinase-1. MMP-9: Matrix metalloproteinase-9. TGF-β: Transforming growth factor beta. TNF-α: Tumour necrosis factor alpha. TNF-β: Tumour necrosis factor beta. TT: Total thiol. NS: non-significant.

**Table 5:** Predictive performance for serum markers to distinguish patients who developed confirmed acute respiratory distress syndrome from mild to moderate pneumonia patients.

Parameter	Sensitivity (%)	Specificity (%)	AUC	95% CI	*p* Value
LDH ^a^ (U/L)	89	73	0.89	0.80–0.97	0
CRP ^a^ (mg/L)	55.6	80	0.67	0.58–0.85	0.221
Ferritin ^a^ (ng/mL)	100	90	0.86	0.80–1.00	0.001
Leukocyte count ^b^	84.6	77.8	0.85	0.75–0.95	0
LDH ^b^ (U/L)	46.2	93.5	0.71	0.57–0.85	0.008
D-dimer ^b^ (ng/mL)	88.5	73.1	0.86	0.75–0.96	0
Ferritin ^b^ (ng/mL)	87.5	81.2	0.91	0.83–1.00	0
AGE (ng/mL)	77.8	65.5	0.77	0.64–0.89	0.001
IL-6 (pg/mL)	79.3	80.6	0.87	0.79–0.96	0
MMP-9 (µg/L)	84.6	73.3	0.82	0.71–0.93	0
TNF-α (ng/L)	48.3	77.4	0.66	0.52–0.80	0.031
TT (µM)	86	85	0.92	0.85–0.99	0

AUC: Area under curve. ^a^: measured on hospital admission. ^b^: measured a week after hospital admission. LDH: Lactate dehydrogenase. CRP: C-reactive protein. AGE: Advanced glycosylated end products. IL-6: Interleukin 6. MMP-9: Matrix metalloproteinase-9. TNF-α: Tumour necrosis factor alpha. TT: Total thiol.

## Data Availability

Data is contained within the article.

## Supplementary Materials

The following are available online at https://www.mdpi.com/article/10.3390/antiox10081221/s1, Figure S1: ROC figure for the significant parameters presented in Table 5, Figure S2: Evolution of biomarkers in ARDS and non-ARDS patients between admission and at 1 week, Table S1: Multiple regression of the different biomarkers with sex and smoking, Table S2: Correlation coefficients between the total thiols and biomarkers.
