# Supplementary material for: Acute Lung Injury Biomarkers in the Prediction of COVID-19 Severity: Total Thiol, Ferritin and Lactate Dehydrogenase
Source: Antioxidants (Basel). 2021 Jul 29;10(8):1221. doi: 10.3390/antiox10081221 (PMC8388961; doi:10.3390/antiox10081221)
Supplement: Supplementary file 1 [file antioxidants-10-01221-s001.zip › antioxidants-1277044-supplementary.pdf]

antioxidants MDPI

# SUPPLEMENTARY

## Acute lung injury biomarkers in the prediction of COVID-19 severity: total thiol, ferritin and lactate dehydrogenase

**Alvaro Martinez Mesa [1], Eva Cabrera César [1, *,†] Elisa Martín-Montañez [2, †], Esther Sanchez Alvarez [1], Pilar Martinez Lopez [1], Yanina Romero-Zerbo [3], Maria Inmaculada Garcia-Fernandez [3, †] and Jose Luis Velasco Garrido[1,†]**

**Correspondence: evacabreracesar@gmail.com**

**Table S1. Multiple regression of the different biomarkers with sex and smoking.**

| **Variable** | **df** | **F** | **p** | **R²** |
|---|---|---|---|---|
| LDH admission | 2, 57 | 1,083 | 0,346 | 0,063 |
| PCR admission | 2,57 | 2,921 | 0,063 | 0,099 |
| Ferritin admission | 2,57 | 3,785 | 0,038 | 0,215 |
| Leukocytes | 2, 57 | 2,198 | 0,122 | 0,081 |
| LDH | 2, 57 | 1,926 | 0,156 | 0,067 |
| D-dimer | 2, 57 | 0,339 | 0,998 | 0,014 |
| Ferritin | 2, 57 | 5,528 | 0,008 | 0,230 |
| AGE | 2, 57 | 1,474 | 0,238 | 0,049 |
| RAGE | 2, 57 | 0,019 | 0,998 | 0,000 |
| IL-6 | 2, 57 | 3,933 | 0,025 | 0,121 |
| MPP-9 | 2, 57 | 0,019 | 0,998 | 0,000 |
| TNF-α | 2, 57 | 1,221 | 0,303 | 0,041 |
| TT | 2, 57 | 1,107 | 0,338 | 0,041 |

A multiple regression was run to predict at admission (LDH, PCR, Ferritin) and after 1week (Leukocytes, LDH, D-dimer, Ferritin, AGE, RAGE, IL-6, MPP-9, TNF-α TT), from gender and smoking status. We were shown that only for IL-6, Ferritin (admission and after 1 week) smoking variable added statistically significantly differences to the prediction, $p < 0.05$. Df: degree of freedom, F: Good of fit value, p statistic value; $R^2$: coefficient of determination.

**Table S2. Correlation coefficients between the total thiols and biomarkers.**

| **Variable pair TT vs** | **R (p)** |
|---|---|
| D-dimer 1 week | -0.566 (0.000) |
| LDH admission | -0.433 (0.002) |
| IL-6 | -0.424 (0.001) |
| TNF-$\alpha$ | -0.366 (0.006) |
| RAGE | -0.344 (0.010) |
| Leukocytes count 1 week | -0.444 (0.001 |

Rho Sperman correlation test was used. n=60. R: correlation coefficient.

## Figure S1. ROC figure for the significant parameters presented in table 5:

**1a) CRP ng/mL admission**

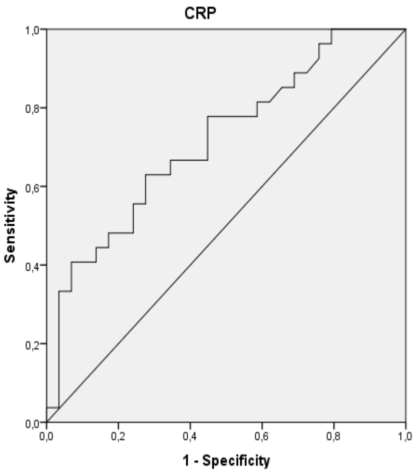


**1b) Ferrin ng/mL admission**

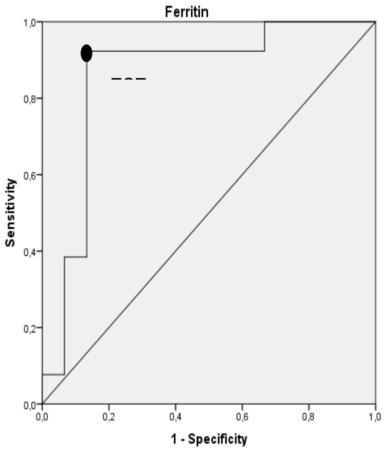


**1c) Leukocytes count (1 week)**

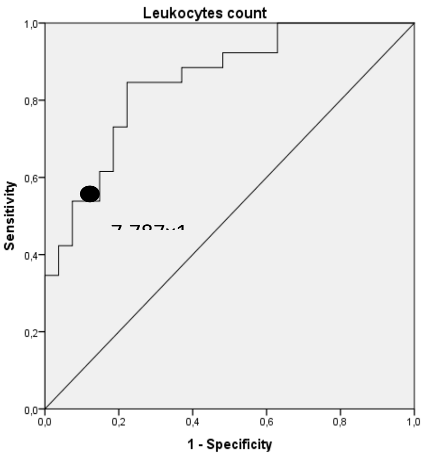


**1d) LDH U/L ( 1 week)**

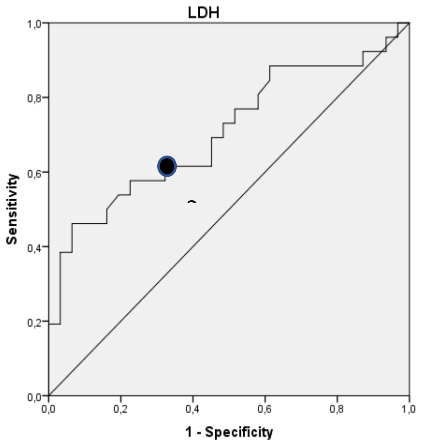


**1e) D-dimer ng/mL(1 week)**

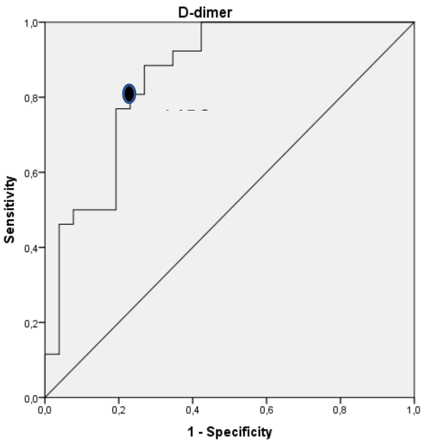


**1f) MMP-9 µg/L**

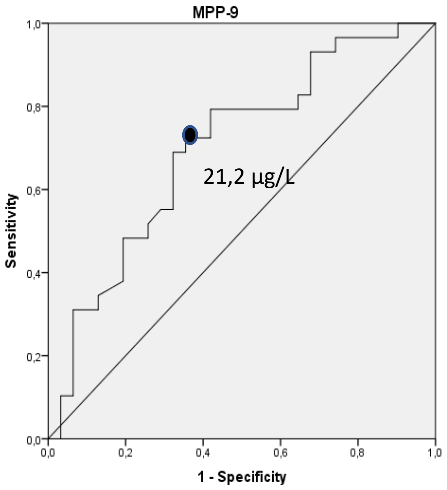

**1g) TNF-$\alpha$ µg/L**

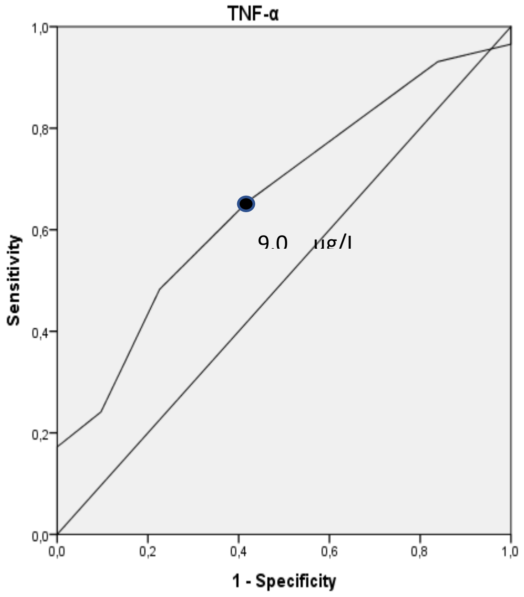


**1h) AGE ng/mL**

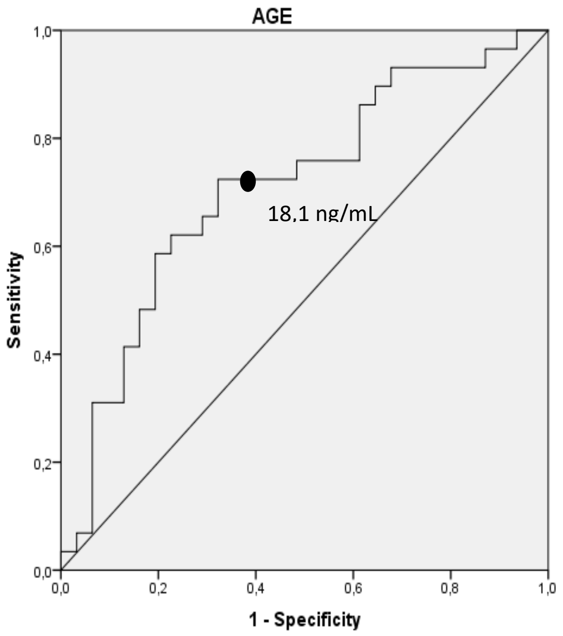


**1i) IL-6 pg/mL**

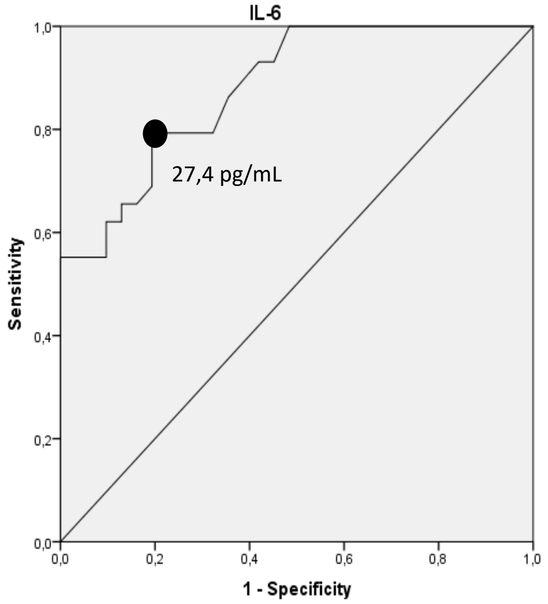

**Figure S2. Evolution of biomarkers in ARDS and non-ARDS patients between admission and at 1 week.**

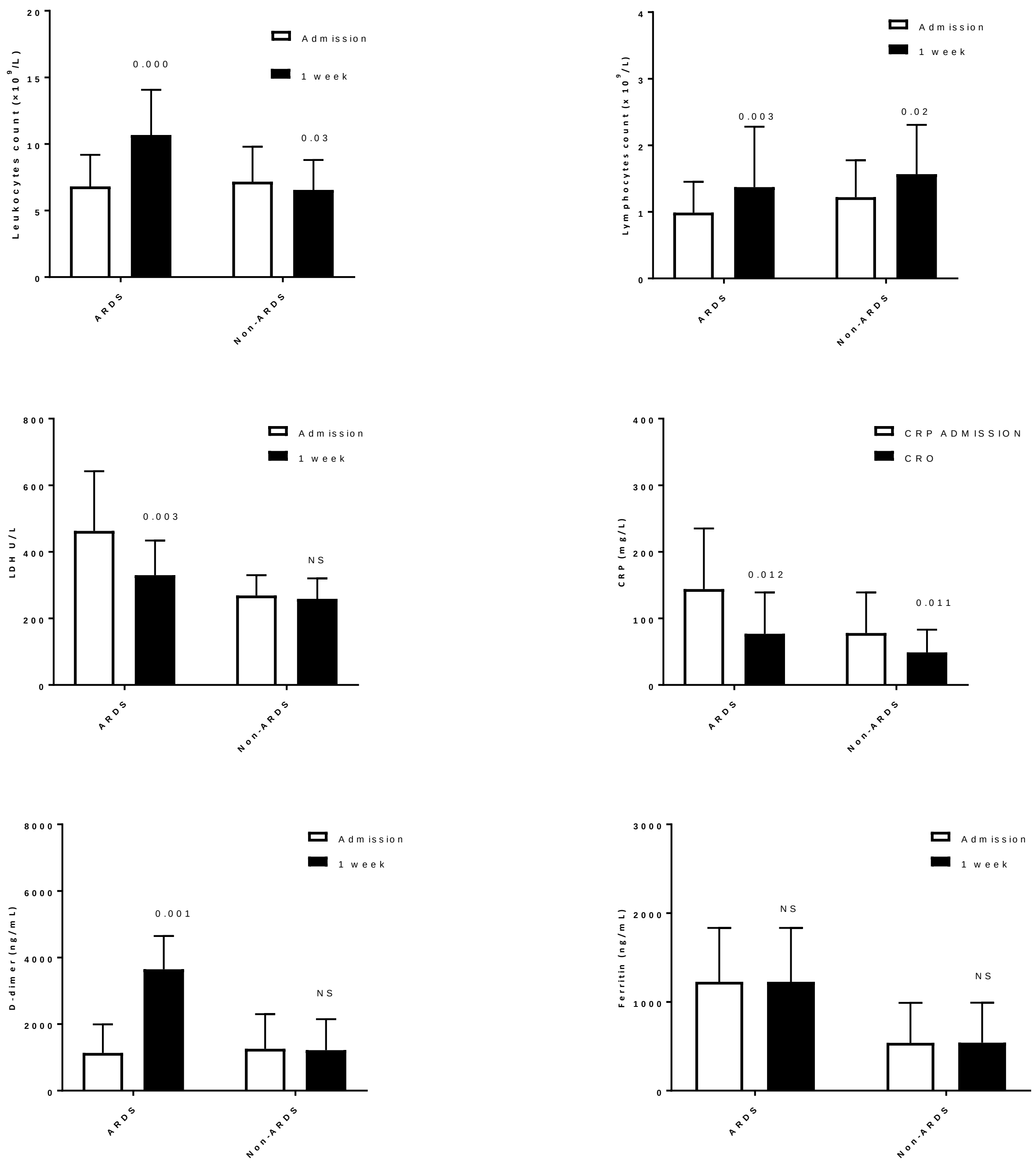


Wilcoxon signed-rank test was used to compare two related samples. ADRS (n=31) and Non-ADRS (n=29). The statistically significant are showed in the top of box.
